# A Robust UWSN Handover Prediction System Using Ensemble Learning

**DOI:** 10.3390/s21175777

**Published:** 2021-08-27

**Authors:** Esraa Eldesouky, Mahmoud Bekhit, Ahmed Fathalla, Ahmad Salah, Ahmed Ali

**Affiliations:** 1 Department of Computer Science, College of Computer Engineering and Sciences, Prince Sattam Bin Abdulaziz University, Al-Kharj 11942, Saudi Arabia; em.eldesouky@psau.edu.sa (E.E.); a.abdalrahman@psau.edu.sa (A.A.); 2 Computer Science Department, Faculty of Computers and Informatics, Suez Canal University, Ismailia 41522, Egypt; 3 School of Electrical and Data Engineering, University of Technology Sydney, Sydney 2007, Australia; mahmoud_bakhit@science.suez.edu.eg; 4 Department of Mathematics, Faculty of Science, Suez Canal University, Ismailia 41522, Egypt; 5 College of Information Science and Engineering, Hunan University, Changsha 410082, China; 6 Faculty of Computers and Informatics, Zagazig University, Sharkeya 44523, Egypt; 7 Higher Future Institute for Specialized Technological Studies, Cairo 3044, Egypt

**Keywords:** ensemble learning, gradient boost, handover prediction, machine learning, sea buoys, underwater wireless sensor networks

## Abstract

The use of underwater wireless sensor networks (UWSNs) for collaborative monitoring and marine data collection tasks is rapidly increasing. One of the major challenges associated with building these networks is handover prediction; this is because the mobility model of the sensor nodes is different from that of ground-based wireless sensor network (WSN) devices. Therefore, handover prediction is the focus of the present work. There have been limited efforts in addressing the handover prediction problem in UWSNs and in the use of ensemble learning in handover prediction for UWSNs. Hence, we propose the simulation of the sensor node mobility using real marine data collected by the Korea Hydrographic and Oceanographic Agency. These data include the water current speed and direction between data. The proposed simulation consists of a large number of sensor nodes and base stations in a UWSN. Next, we collected the handover events from the simulation, which were utilized as a dataset for the handover prediction task. Finally, we utilized four machine learning prediction algorithms (i.e., gradient boosting, decision tree (DT), Gaussian naive Bayes (GNB), and K-nearest neighbor (KNN)) to predict handover events based on historically collected handover events. The obtained prediction accuracy rates were above 95%. The best prediction accuracy rate achieved by the state-of-the-art method was 56% for any UWSN. Moreover, when the proposed models were evaluated on performance metrics, the measured evolution scores emphasized the high quality of the proposed prediction models. While the ensemble learning model outperformed the GNB and KNN models, the performance of ensemble learning and decision tree models was almost identical.

## 1. Introduction

With the dramatic growth in marine monitoring (especially oceans), recently developed technologies must be deployed in UWSNs, also called acoustic networks, to obtain environmental details. These network-based monitoring systems consist of collaborative underwater sensors that can communicate, capture, and process data [1]. However, the highly dynamic nature of UWSNs increases the node mobility due to water currents. Underwater artifacts can travel at 2–3 knots or 3–6 km/h in a normal underwater state at a speed following empirical observations [2]. This speed hinders the coherent tracking of such sensor locations or allocation to the nearest buoy. A robust handover process could ensure continuous and consistent timely data transmission over the network and achieve uninterrupted communication.

Several proposed handover prediction methods utilize machine learning models (e.g., [3,4]) to obtain better communication on terrestrial sensor networks. Other works applying machine learning models to improve the handover prediction problem in UWSNs include [1,5]. The authors in [3] developed a new technique to predict blind spots and handover associated with the movement of Wi-Fi devices, where video buffers are stuffed with low-resolution video frames before resources are reallocated or the connection is lost. The proposed prediction method is based on a machine learning system, where the pattern of the received signal strength indicator (RSSI) is used to determine a future handover over time. In [4], the authors proposed two different methods to predict the handovers of a mobile user using the user’s behavior information given by the networks. To validate their method, the authors compared the performance of the proposed handover prediction mechanism to decision tree classification and K-nearest neighbors.

In the UWSN model, sensor nodes usually move by themselves (i.e., autonomous underwater vehicles) [6] or passively move due to flows and other natural impacts [7]. It is very difficult to define and control the locations of passive nodes. The authors in [5] proposed machine learning models to predict the flow directions of the sensor nodes that flowed in the surface layers of the ocean. In [1], the authors proposed a new model based on machine learning techniques for handover prediction problems in the Internet of Underwater Things (IoUT). The prediction model removes the overhead associated with channel measurement to reduce the power consumed by underwater things. In [1,5], the obtained prediction accuracy rates were very low (approximately 56%). In addition to the low prediction accuracy of the existing methods, the authors did not make the dataset publicly available. Thus, it is impossible to reproduce the results reported in [1,5].

The dynamicity and continuous flow of sensor nodes make them shift away from their locations toward other sectors. When this occurs, successful and timely handover is vital to guide the sensor node to the nearest sea buoy in order to guarantee efficient and uninterrupted data transmission. However, existing acoustic handover mechanisms are not feasible due to their long propagation time, unreliable sensor node location, and poor transmission rates. Since machine learning techniques are rarely used in handover prediction in UWSNs, we are motivated to utilize ensemble learning (e.g., gradient boosting) to predict handover events in UWSNs, as ensemble learning has not been used to address this problem.

In this work, we simulate the movement of UWSN sensor nodes using real open ocean marine data such as water current speed and direction. We utilize real-time data provided by the Korea Hydrographic and Oceanographic Agency (https://www.khoa.go.kr/eng/ (accessed on 26 August 2021)) for this purpose. This agency monitors the southeastern coasts of the Korean Peninsula [1,5]. The simulation captures the handover events, which are used as a dataset for the proposed work. Finally, we utilize several machine learning methods to predict the future coverage cell for the sensor nodes in handover events. Finally, we utilize several machine learning methods, including DT, GNB, KNN, and gradient boosting to predict the future coverage cell for the sensor nodes in handover events. The major contributions of this paper can be summarized as follows.

- To the best of our knowledge, this is the first work that provides a handover dataset created from real open ocean marine data (https://github.com/Ahmed-Fathalla/UWSN (accessed on 26 August 2021)). This is also the first work to provide the utilized datasets and source code in the field of UWSN handover prediction; thus, the reported results are reproducible.
- To the best of our knowledge, this is the first effort to employ an ensemble learning approach in a UWSN that explores the handover prediction of sensor nodes.
- The proposed handover prediction models outperform the existing state-of-the-art methods with a huge performance gap.

The remainder of the paper is organized as follows. The background of this work is discussed in Section 2. Section 3 discusses the state-of-the-art methods. In Section 4, we explain the proposed handover prediction system. Section 5 discusses the proposed experiments and obtained results. Finally, the paper is concluded in Section 6.

## 2. Background

Machine learning and its applications have proven their success in many applications [8,9,10]. Therefore, in this paper, several machine learning models have been utilized, including DT, GNB, KNN, and gradient boosting. In this section, we explain the background of each of these models.

### 2.1. Decision Tree

The DT model is currently one of the most widely used mechanisms in several applications for predictive modeling, including regression and classification. The decision tree algorithm works very well and continuously if the data are discontinuous, even if noise appears. Moreover, it can handle collinearity efficiently and provide excellent prediction explanation. On the other hand, DTs suffer from higher complexity, especially when dealing with complicated datasets, and consequently may lose valuable information (i.e., in the case of continuous variables).

Several techniques have been used to determine the best way to split the input data. One of the main goals of the DT model is to find the most significant splits between tree nodes and further optimize the data into correct classifications. To achieve this goal, it is necessary to apply proper decision rules to the proposed data, which significantly affects the algorithm’s performance.

Information gain (IG) is used with the DT model. The decision tree model aims to find the best split node that guarantees high accuracy. The IG method seeks to find the most suitable nodes that return the highest information gain, which can be measured using an entropy factor. The entropy factor is used to determine the degree of disorganization in the system. The entropy for the output can be calculated using the following formula:

(1) $$E(s)=\sum_{i=1}^{c}-p_{i}\log_{2}p_{i}.$$

### 2.2. Gaussian Naive Bayes

The GNB model is considered a conditional probability model (i.e., the probability that an event will occur if another event occurs), and the information about the latter event is provided to the model. Using the conditional probability and the information provided by previous events, the probability of a future event can be obtained using Equation (2):

(2) $$P\left(A|B\right)=\frac{P(B|A)P(A)}{P(B)}$$

where P(A|B) is a posterior probability and represents the likelihood of *A* by the value of *B*; P(B|A) represents the probability of *B* given a value of *A*; P(A) is the prior probability and represents the probability of event *A*; and P(B) is a marginal probability and represents the probability of event *B*. Using Bayes’ theorem as a basis, the GNB can be formulated as follows:

(3) $$P\left(y|x_{1},...,x_{j}\right)=\frac{P\left(x_{1},...,x_{j}|y\right)P\left(y\right)}{P(x_{1},...,x_{j})}$$

where $P(y|x_{1},...,x_{j})$ is a posterior probability and represents the data probability of the model including class *y* given the feature value from $x_{1}$ to xj. $P(x_{1},...,x_{j}|y)$ is the likelihood of feature values given their *y* class. P(y) is a prior probability, and $P(x_{1}...,x_{j})$ is a marginal probability.

The GNB is a generative model and is considered a simple and a powerful model that can be used in solving prediction problems. In addition, this model can utilize less data for training. However, the GNB has limited use in the real world as it assumes all features are independent.

### 2.3. K-Nearest Neighbor

The KNN model is one of the simplest and most straightforward supervised learning models in machine learning. The key idea of this algorithm is to decide a predicted value based on the labeled data points of the training set that are near the query data point. KNN starts by loading the training data points in memory. Then, the classification task is completed by finding the nearest *K* data points. Finally, a vote of the *K* closest points to the query point will determine the class of the query data point. One critical decision that needs to be made is the selection of the distance function. Several distance functions have been proposed to compute the distance between two data points; however, the most common methods are cosine similarity and Euclidean distance.

The Euclidean distance can be calculated by subtracting the training data point from the point to be classified, as in Equation (4):

(4) $$E(x,y)=\sqrt{\sum_{i=0}^{n}{\left(x_{i}-y_{j}\right)}^{2}.}$$

The calculation of the marginal probability can be ignored in this model, as it has the same value as in the GNB calculations. In this calculation, we determine the data point class based on the best posterior probability value. Although KNN contains a limited number of hyperparameters (i.e., the k-value and distance function), which makes it a simple model, the *K*-value can dramatically affect the model performance. Similar to DT, the KNN model has a large computation cost if the dataset is large.

### 2.4. Gradient Boosting

Boosting is a well-known weighted ensemble method in which each base algorithm is sequentially implemented. The weights are modified and updated to place a greater emphasis on training tuples, especially those that were misclassified by the previous classifier. Gradient boosting is a subset of boosting where the loss function is minimized using DT as a weak learner, as well as gradient descent. Additionally, the loss function is employed to enhance the performance of the learner in subsequent iterations. Typically, the relative weights of the previous misclassified tuples are modified in each iteration of the boosting classifier. As a result, the boosting classifier, which is based on the reinforcement learning principle [7], is extremely robust. In this work, we focus on the use of gradient boosting in decision trees.

Various problems, such as regression, multiclass classification, and ranking, can be resolved using gradient boosting [11]. Gradient boosting outperforms other classification algorithms with regard to processing categorical features (i.e., a distinct set of incomparable values) while maintaining high accuracy. Categorical features in gradient boosting are handled at the preprocessing phase, which entails substituting one or more numerical values for the original categorical variables. In addition, missing value support, ordered boosting implementation, ease of using graphics processing unit (GPU) training, and visualization are significant characteristics of gradient boosting. It also enables the estimation of leaf values using random permutations while selecting the tree structure (i.e., binary decision tree) to avoid the overfitting associated with the gradient algorithms.

## 3. Literature Review

In this section, we highlight the recent handover mechanisms in wireless networks, which mainly deploy prediction approaches (e.g., classification models). Then, we address the basic problems associated with UWSNs and list efforts directed to their applications. Finally, we narrow this literature review to describe handover problems in UWSNs.

In the last few years, several studies have proposed solutions to enable smooth mobility in wireless networks by improving the handover process. In [12], authors proposed two prediction mechanisms to estimate mobile user handovers using user–network association patterns. They conducted a series of experiments using real data to compare the performance of such mechanisms against more advanced and complex prediction systems. In another study, the handover process of Wi-Fi networks was predicted using a novel technique [3]. The primary purpose of this technique is to maintain the connections between resources when they are reallocated or fill out the video buffer with low-definition video frames before the user loses the connection. In addition, a recent study proposed clustering and classifying algorithms to streamline the 5G handover procedure and enhance the network connectivity [4].

**Figure 1:**
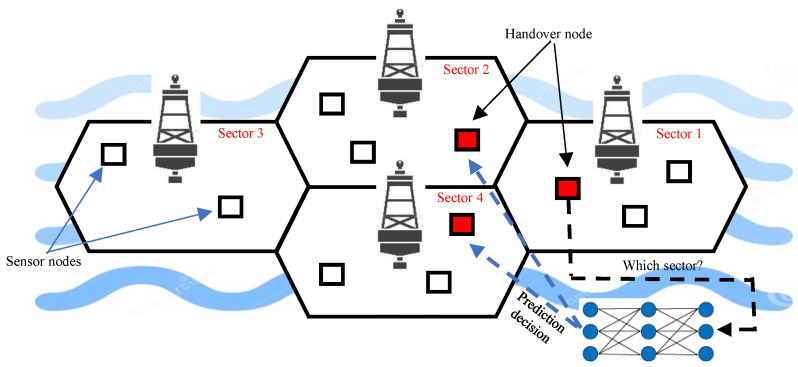
Schematic diagram of the use of machine learning models to predict the handover of a sensor node in a UWSN.

A UWSN consists of a set of sensor nodes placed in a specific marine area which communicate over an acoustic channel, as shown in Figure 1. This communication can be established under restricted circumstances with a possibility of long propagation delay and high bit error rates, considering the restrictions of batteries [13,14]. In [15], the authors proposed a new technique to correct the bit error based on energy analysis and considering the network conditions.

Recently, numerous research papers have developed solutions to improve underwater applications and ocean exploration needs. One of the most critical applications is the monitoring of the physical environment (e.g., battlefields, floods, volcanoes), and other applications that rely on data from these sensors [16,17].

Additionally, interest in underwater communication systems for ocean observation has dramatically grown over the last decade. Through ocean monitoring, many applications are addressed, including military plans, marine weather forecasting, oceanographic data compilation, and environmental observation [18]. These applications involve autonomous underwater vehicle (AUV) communications, oil extraction, ocean pollution, and aquaculture [19,20]. AUVs, sea buoys, and sensor nodes are positioned in a geographic region to collaboratively monitor and gather data in underwater sensor networks. Currently, a higher bandwidth with adequate communication schemes is required to accommodate the deployment of AUVs and sensors that continuously collect real-time data (i.e., images, videos, water temperature, and salinity). However, the lack of interactive communication among installed devices can impede data delivery and lead to a loss of the collected data.

A new approach was presented in [21] to expand the handover protocol in clustered ad hoc diver networks to enhance the reliability and flexibility of the connections. Moreover, a new method was implemented and tested with different nodes to allow multi-hop connections using a low-cost solar-powered underwater modem in [22]. The proposed method checks whether the diver leaves the communication range; if so, the model applies the handover process by using the diver as a bridge between the receiver and the transmitter. The model presents a suggestion for the handover concepts to be applied only and does not provide an experiment or investigate the proposed idea. Additionally, the proposed method does not consider placing any restrictions on the distance between nodes, since the model uses visual light.

Nevertheless, efficient determination of the positions of the sensor nodes and their signal intensity is a crucial challenge in UWSNs. The sensor nodes must be attached to the appropriate buoy to ensure constant data delivery [23]. Another critical issue that affects the data gathering and forwarding operations between sensor nodes and buoys is the successful handover in underwater environments. Unlike handovers in ground-based WSNs, the delay time in UWSNs is longer, and it is more difficult to test signal strength. According to recent studies [1,5,24], handover in UWSNs solely depends on location-based potentials. The cited works do not provide their datasets nor their source code; thus, it is impossible to reproduce their experiments. The use of any machine learning classifier model (e.g., decision tree) with different parameter values results in different accuracy rates. The authors of [1,5,24] did not discuss the parameters of the utilized machine learning models. Moreover, the obtained handover prediction scores are considered very low: less than 56% for the top rank 1 prediction.

To the best of our knowledge, handover prediction techniques for UWSNs utilizing marine data have only been proposed in [1,5,24]. This lack of previous research and standards leads to an unsystematic system for researchers in the future. This paper is the first attempt to address the solution of the handover problem using ensemble learning for large UWSNs, as shown in Figure 1.

## 4. The Proposed System

### 4.1. An Overview

**Figure 2:**
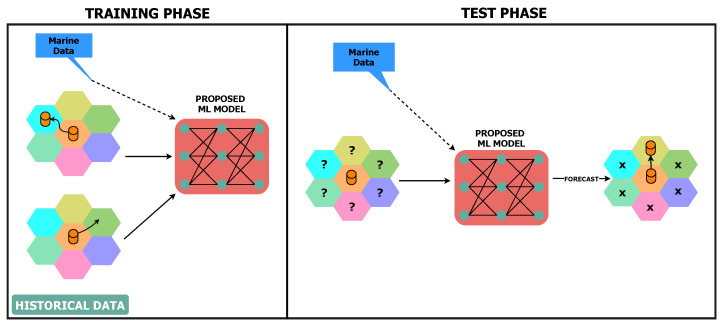
Overview of the proposed system.

**Figure 3:**
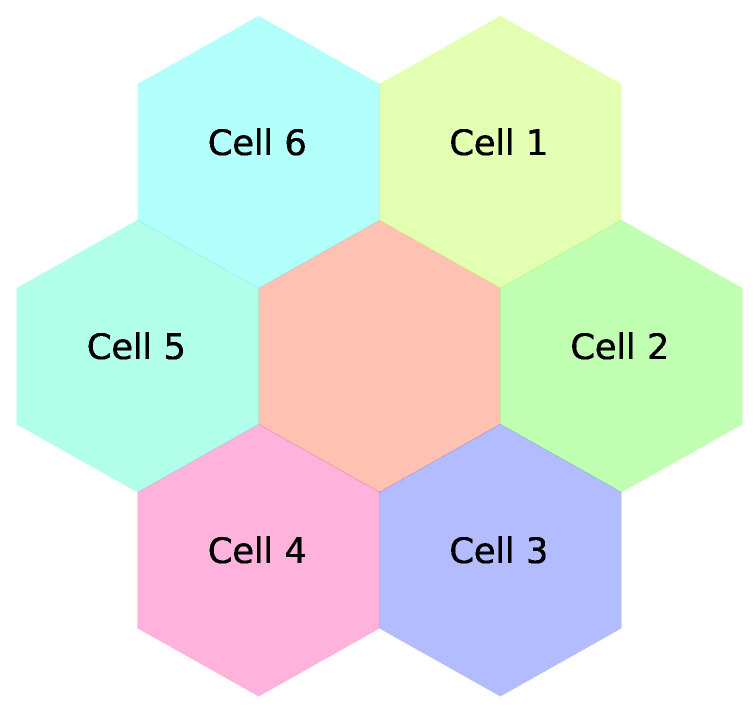
An example for a cell with its six neighboring cells.

**Figure 4:**
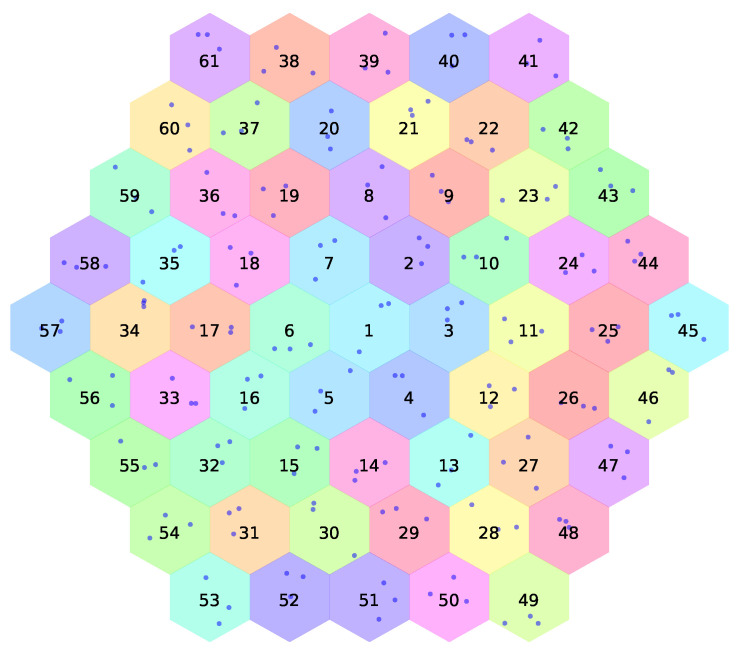
The proposed simulated UWSN with 61 base stations.

The UWSN in the proposed work consists of a set of sensor nodes and sea buoys that are replaced in a specified area (Figure 1). In this non-static network where node mobility is presented due to the continuous water currents, sensor nodes can move far from their sea buoy (cell centroid). This consequently can lead to the loss of connection between the sensor node and its sea buoy, which requires a successful handover process to help the sensor node to safely attach to a new sea buoy. According to Figure 2, the classifier initiates a training phase based on historical data (i.e., from the Korea Hydrographic and Oceanographic Agency dataset). The proposed ML model considers the sensor’s location, the received signal strength from neighboring buoys, and the weather forecast to predict the suitable buoy to complete the handover process successfully. Finally, in the test phase, the proposed model assists the sensor node in making adequate handover decisions. The structure of the cells is illustrated in Figure 3 and Figure 4.

**Figure 5:**
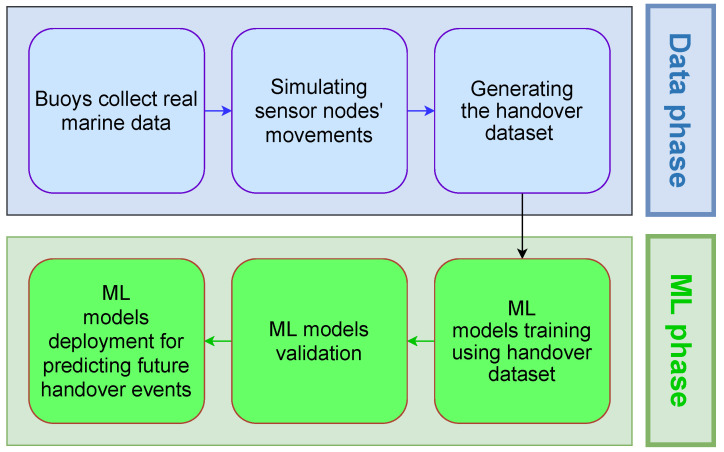
Block diagram of the proposed system.

Figure 5 depicts a block diagram of the proposed UWSN handover prediction system. The proposed system consists of two phases: the data collection phase and the machine learning phase. The data collection phase consists of collecting the handover occurrence data. That is, when a handover occurs in the network, the sensor sends environmental data (e.g., sensor location and signal strength of neighbor cells) of the last collected data before the handover. The second phase (the machine learning phase) is responsible for training, validating the machine learning models using handover data, and starting the deployment of the UWSN handover prediction system.

### 4.2. Dataset Generation

The Korea Hydrographic and Oceanographic Agency collects a public real-time dataset for information observation, which is updated every 30 min, as previously mentioned. The underwater network is located at 34.223611 latitude and 128.420555 longitude (https://www.khoa.go.kr/eng/ (accessed on 26 August 2021)). The region is divided into sectors by centroid sea buoys, which relay data from nearby underwater sensors to the aboveground base station. Prior to transmission, relevant information is processed by the buoys, which includes measurements of the time, water salinity, water temperature, wave height, sea surface direction, and flow speed. The aforementioned dataset is extremely rich with vital marine information. This information can help to predict the movement of the sensor nodes.

Several existing research works have discussed the mobility of devices and their role in predicting handover events [25,26]. Most of these existing methods target on-land handover prediction. A few exceptions discuss UWSN mobility models [27]. Underwater sensor nodes are not stationary. Instead, they travel with water currents a result of various activities and conditions present underwater. Thus, in this work we use real marine data from the Korea Hydrographic and Oceanographic Agency to simulate the sensor node movements. Then, the captures the movement of these sensor nodes, which is used as a history of handover events. Finally, the captured handover events is utilized to train several machine learning models to predict the next covering cell of a sensor node in a handover event.

Underwater sensor network nodes can be static or non-static, similar to ground-based sensor network nodes [28]. The latter type moves with water currents due to different activities and circumstances in the underwater environment. This work focuses on non-static sensor nodes. Figure 3 depicts a buoy that is surrounded by six different cells (i.e., buoys). Each buoy of the Korea Hydrographic and Oceanographic Agency is represented by a hexagon that shows its coverage area. The simulation consists of a set of buoys, each of which has a number of sensor nodes (i.e., dots), as shown in Figure 4. The movement of each sensor node is based on the marine data collected by each buoy.

The simulation starts by initially placing the sensor nodes in the coverage area of each buoy. Then, these sensor nodes wag in specific directions with the guidance of the velocity and direction of the water currents. As a result, the data in the dataset aid in sensor guidance by successfully recommending a nearby buoy, which results in an efficient handover operation. Uninterrupted data transmission from sensor nodes is assured in this manner.

During the movements of the sensor nodes, the simulation captures several pieces of information, that is, the sensor node location in terms of latitude and longitude, the ID of the buoy that provides the coverage, and the signal strength. During the simulation, the sensor node location is updated based on the water speed and direction using Equations (5) and (6) [27].

(5) $$X_{t+1}=X_{t}+\alpha \times s\times \sin\left(dir\right)$$

(6) $$Y_{t+1}=Y_{t}+\alpha \times s\times \cos\left(dir\right)$$

where $X_{t+1}$ and $Y_{t+1}$ are the next latitude and longitude of the sensor node, respectively, and $X_{t}$ and $Y_{t}$ are the current latitude and longitude of the sensor node, respectively. α is a parameter to tune the speed of the sensor node based on the level at which the sensor node is located under the water surface; if α=1, the sensor node moves at the full speed of the water. *s* and dir are the speed and direction of the water, respectively. Finally, the sin and cos functions are from Newton’s second law of motion. Each node has a signal strength, which is measured by the Friis transmission equation (Equation (7)) as follows:

(7) $$P_{r}=P_{t}G_{t}G_{r}{\left(\frac{\lambda }{4\pi d}\right)}^{2}$$

where $P_{r}$ and $P_{t}$ are the signal power of the receiver (i.e., sensor node) and transmitter (i.e., the buoy), respectively; $G_{r}$ and $G_{t}$ are the antenna gains of the receiver and transmitter, respectively; *d* represents the distance between the receiver and the transmitter; and λ is the wavelength.

### 4.3. Evaluation Metrics

During the classification process, the evaluation metric is crucial for discriminating and obtaining an optimal classifier. Hence, five essential metrics are considered in this paper. Recall (*r*) is one of the metrics to find the percentage of the correctly classified positive pattern that the classifier should collect. Precision (*p*) determines the fraction of positive patterns in a positive class that are correctly predicted out of the overall predicted patterns. Both terms are often used to assess the goodness of a model in predicting observed instances [29,30]. Recall and precision are defined in Equations (8) and (9), respectively:

(8) $$r=\frac{t_{p}}{t_{p}+t_{n}}$$

(9) $$p=\frac{t_{p}}{t_{p}+f_{p}}$$

where $t_{p}$ and $t_{n}$ are the positive and negative instances that are correctly classified, respectively, and $f_{p}$ is the number of misclassified positive patterns.

Recall and precision are known to detect the sensitivity and positive predictive value of a classifier, respectively. However, they cannot be individually employed to evaluate the handover prediction in the proposed simulation. In that, the F-score metric (*F*) is the harmonic mean between recall and precision values. Further, the F-score combines both metrics to obtain a scalar value as in Equation (10).

(10) $$F1-score=\frac{2pr}{p+r}$$

Another related metric is the accuracy (*A*), which is defined as the proportion of correct predictions to all the predictions conducted (i.e., the total number of instances calculated). While the accuracy is a significant metric, it cannot be used alone to determine the robustness of the classifier. The accuracy (*A*) metric is calculated as shown in Equation (11).

(11) $$A=\frac{t_{p}+t_{n}}{t_{p}+f_{p}+t_{n}+f_{n}}$$

where $f_{n}$ is the number of misclassified negative instances.

Finally, a receiver operating characteristic (ROC) curve is a graphical representation of the success of a classifier across all classification thresholds. The true positive rate and false positive rate are the main parameters that build this graph. An ROC curve can be generated by repeatedly running a logistic regression model. However, an aggregate measure of performance is determined for the overall possible classification thresholds, which is known as the area under the curve (AUC). It is utilized to calculate the 2D area under the ROC curve from (0, 0) to (1, 1).

## 5. Experimental Results

### 5.1. Setup

Table 1 lists the values of the parameters used in the simulation. The parameter values were selected according to [31,32]. We conducted two types of experiments. In the first, we supposed that the sensor node had a Global Positioning System (GPS) unit. Thus, the distance between the sensor node and each buoy in the simulation was known, as the buoys were static to their location. We called this type of experiment Exp-I. In Exp-I, the current location of the sensor node was used as a feature in the prediction model in addition to the other features, which were the signal strength, ID of the current cell, and IDs of the neighboring cells. We called the second type of experiment Exp-II. In Exp-II, we supposed that the location of the sensor node was unknown, and that the sensor node was not equipped with a GPS unit. Thus, the signal strength played a vital role in the handover prediction.

The simulation generated 216,578 different handover events. The collected marine data in the simulation spanned six months (the first six months of 2020). The simulation included 61 buoys and 610 sensor nodes. While the Korea Hydrographic and Oceanographic Agency has only 30 real buoys, the simulation duplicated the marine data of the nonreal buoys from the closest real buoy.

The simulation framework was developed in the Python programming language. Hence, to ensure results reproducibility, we made the simulation framework and the dataset used in this work available online (https://github.com/Ahmed-Fathalla/UWSN (accessed on 26 August 2021)). As mentioned above, the signal strength was used as an effective feature in the handover process. The work in [33,34,35] considered the computation of the received power with different parameter values. However, they did not consider ocean or seawater environments. Thus, the values of the parameters in this experiment relied on the first equation in [31,32]. The proposed system is generic and can handle any network configurations.

### 5.2. Results and Discussion

The proposed system utilized four different classification techniques: decision tres, K-nearest neighbor, Gaussian NB, and gradient boosting. The utilized classification models were evaluated on six different metrics: precision (Equation (9)), recall (Equation (8)), F1-score (Equation (10)), accuracy (Equation (11)), AUC-ROC, and confusion matrix.

Table 2 lists five evaluation metrics for Exp-I and Exp-II for the gradient boosting classifier. The classifiers in Exp-I clearly had a superior overall performance to those in Exp-II because the exact location of the sensor node was one of the input features. If the base station (i.e., the static buoy) and sensor node locations are known, the task of predicting the next cell to serve the sensor node should be easy. The problem becomes more difficult when the handover prediction is based on the signal strength. Thus, the results of Exp-I were higher than those of Exp-II.

**Figure 6:**
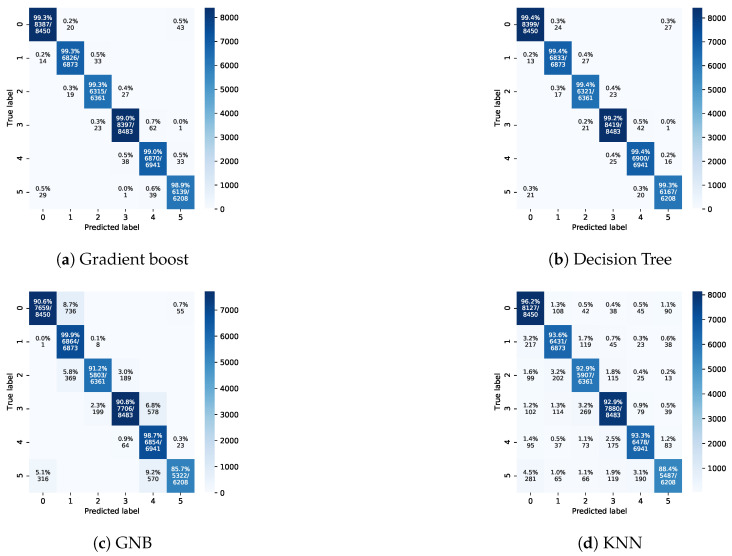
Confusion matrices for Exp-I.

**Figure 7:**
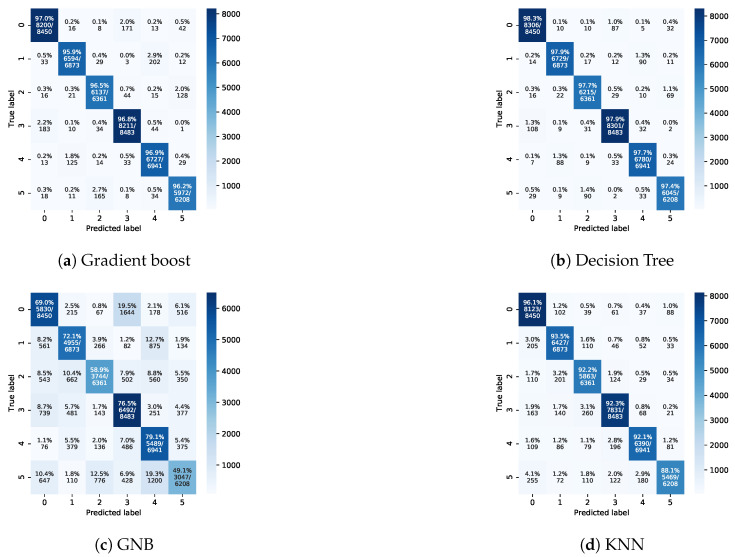
Confusion matrices for Exp-II.

Figure 6 and Figure 7 depict the confusion matrices of the four different classifiers for Exp-I and Exp-II, respectively. The obtained results emphasize the higher results of Exp-I compared to those of Exp-II. In addition, the gradient boosting classifier clearly had the best performance among all classifiers.

**Figure 8:**
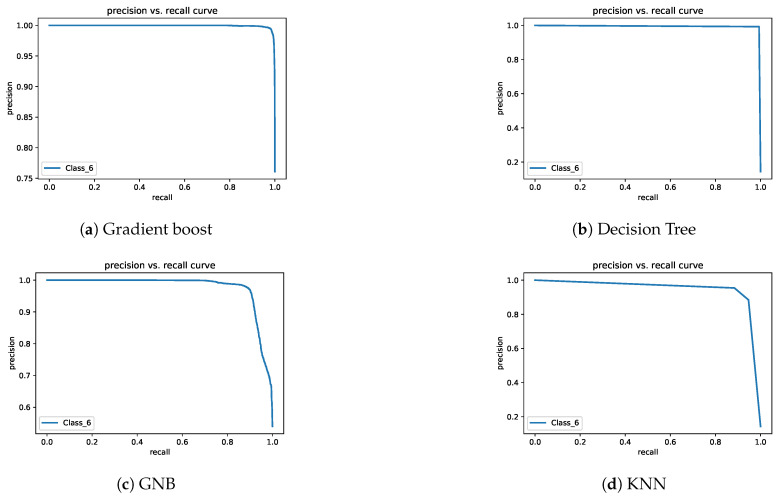
Precision–recall curves for Exp-I.

**Figure 9:**
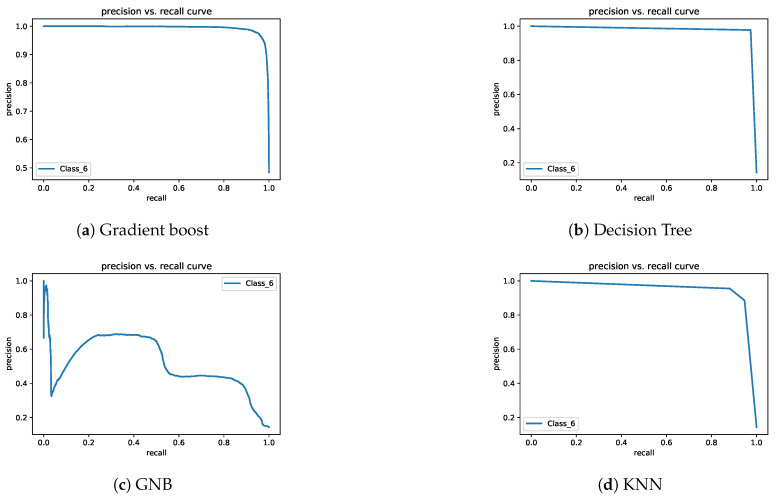
Precision–recall curves for Exp-II.

**Figure 10:**
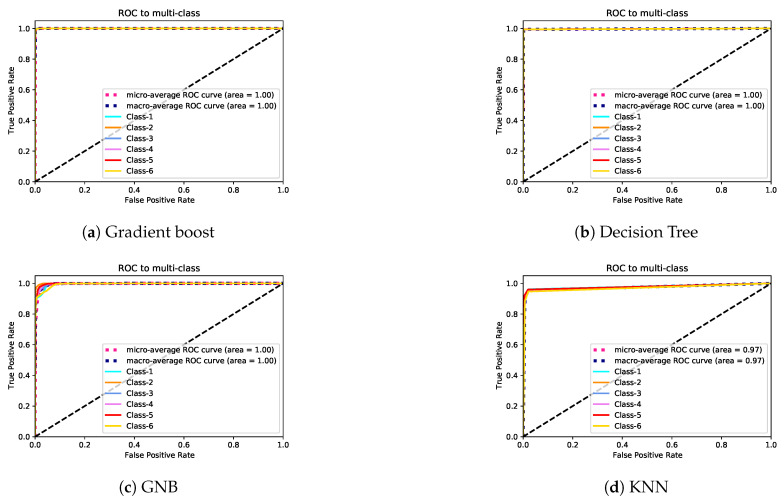
AUC-ROC curves for Exp-I.

In Exp-I and Exp-II, the behaviour of the classes was almost the same for all of the utilized machine learning models except GNB. This behaviour can be noticed in Exp-I (e.g., Figures 7c and 9c) and it is obvious in Exp-II (e.g., Figures 8c and 10c). The lowest performance of GNB can be linked to the assumption of independent predictor features. The features UWSN handover prediction problem is somewhat related to the location of cells and wave directions. Besides, these results indicate that the data of Exp-I were easier to predict in comparison to the data of Exp-II. In Exp-I, the classification rates of the gradient boost and decision tree models were similar. On the other hand, at lower classification rates, the performance of the KNN and GNB models was close. The lowest class accuracy rates were 99.2%, 98.9%, 88.4%, and 85.7% for decision tree, gradient boost, KNN, and GNB, respectively.

**Figure 11:**
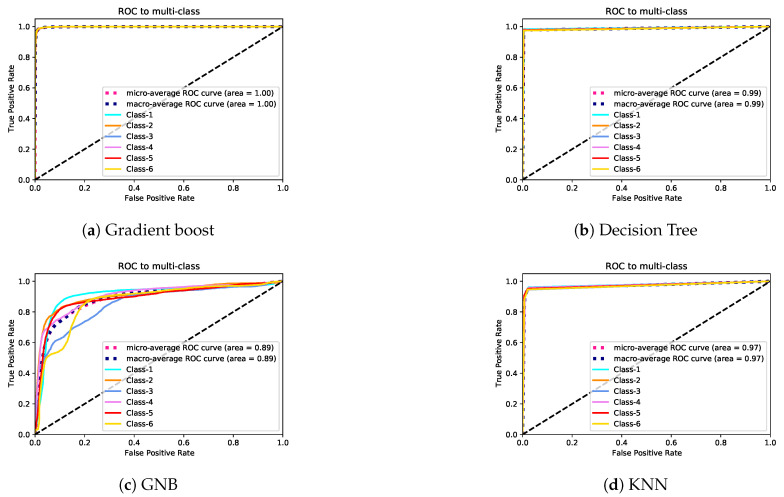
AUC-ROC curves for Exp-II.

In Exp-II, the classification rates of the gradient boost and decision tree models were almost identical. The KNN model’s performance was lower than that of gradient boost and decision tree models. The GNB model’s accuracy was very low. The lowest class accuracy rates were 97.4%, 95.9%, 88.1%, and 49.1% for decision tree, gradient boost, KNN, and GNB, respectively. Figure 8 and Figure 9 depict the precision–recall curves, and Figure 10 and Figure 11 present the AUC-ROC curves. All of these results are consistent with the aforementioned.

The performance of the GNB classifier in Exp-I and Exp-II was the lowest in comparison to the other classifiers. This is linked to the GNB classifier’s assumption that the independence of features should hold true. In this dataset, the input features are dependent to some extent (e.g., the location of the sensor node and the flow direction). The KNN classifier’s performance was higher than that of the GNB classifier and lower than that of the DTs and gradient boost. This is because there are imbalanced data of the classes. For instance, the dataset contains a class/cell with 34,363 handover events, while another class/cell has 31,803 handover events. The KNN classifier’s performance is sensitive to imbalanced data. This data imbalance can be linked to the flow direction. In other words, the flow direction moves the sensors nodes toward certain cells/classes and away from other cells/classes. Thus, the handover events for some classes are greater than for other classes. The performance of the DT and gradient boost classifiers was higher than that of the other two classifiers due to the ability of these two classifiers to handle imbalanced data, and because they do not assume that there is independence of the input features.

## 6. Conclusions

We addressed the problem of handover prediction in UWSNs. Due to the highly dynamic nature of UWSNs, the sensor node mobility model is different from the ground-based mobility model. In addition, the efforts to predict handover events in these networks are very limited. Thus, we proposed the use of marine data to simulate the sensor nodes in the UWSN based on data collected by the Korea Hydrographic and Oceanographic Agency. We collected 216,578 handover events. Finally, we utilized four machine learning models for prediction purposes: gradient boosting, decision tree, Gaussian naive Bayes, and KNN. While the datasets of the state-of-the-art methods [1,5] and the utilized dataset in this work are not identical, both datasets are based on the same marine data (i.e., Korea Hydrographic and Oceanographic Agency data). The obtained prediction accuracy rates were above 95% for the proposed models, which is the highest reported accuracy for the problem of handover prediction in UWSNs. Future research directions include utilizing deep learning methods in the UWSN handover prediction task. This is because the performance of the deep-learning-based models outperform the classic machine-learning-based models, but this comes at the cost of higher training time and larger model size.

## Figures and Tables

**Table 1:** Parameter values of the experiments.

Parameter	Value
α	1
λ	0.125 m
$P_{t}$	5 mW
$G_{r}$	1
$G_{t}$	1

**Table 2:** Average handover prediction results for the gradient boosting model.

Recall	Precision	F1-Score	Accuracy	ROC-AUC
Exp-I				
0.9816	0.9816	0.9816	0.9816	0.9996
Exp-II				
0.9659	0.9659	0.9659	0.9659	0.9987
